# Prevalence of depression among college students living alone in pandemic 2020

**DOI:** 10.1192/j.eurpsy.2021.743

**Published:** 2021-08-13

**Authors:** T. Kantohe, G. Kurnijuanto

**Affiliations:** Faculty Of Medicine, Sam Ratulangi University, Manado, Indonesia

**Keywords:** Pandemic 2020, COVID-19, Depression, College Student

## Abstract

**Introduction:**

The prevalence of depression differs between groups, such as race, gender, and age group. According to the World Health Organization (WHO), depression is one of the leading causes of death in the age group of 15-29 years-old. During the COVID-19 pandemic, some countries, including Indonesia, ordered a nationwide physical distancing and limited public activities. Indonesia also restricts the public mobilities, leaving the college students, studying in different regions, isolated and confined to their flats, boarding houses, and apartments.

**Objectives:**

To find out the prevalence of Depression in College Students, living alone, in Pandemic 2020.

**Methods:**

College students aged 18 to 28 years old, were assessed using Beck’s Depression Inventory (BDI-II), through Google Form, and shared using social media. We also grouped the participants to age, gender, religion, and types of housing, and did the multivariate analysis using median comparison and multinomial logistic regression.

**Results:**

A total of 84 college students, who are living alone during the Pandemic 2020, participated in this study. We found that 33.3% of the population suffered from depression. The median score for the group of females, prefer not to say their religion, and living in the owned house have a higher median. While multinomial logistic regression study does not have any significant odd risks in the variables.

**Conclusions:**

The results indicate that in time of nationwide physical distancing and limited public activities due to COVID-19 Pandemic, the prevalence of depression among college students living alone in Pandemic 2020 is 33%. One-fourth of them are with severe depression.

